# Human-AI collaborative generative AI for comfortable care: a doctor–patient–AI feedback loop for pre-operative distress and workforce shortages

**DOI:** 10.3389/fpubh.2026.2001218

**Published:** 2026-10-28

**Authors:** Hui Sun, Yan Luan, Bo Song

**Affiliations:** Yantaishan Hospital, Yantai, Shandong, China

**Keywords:** clinician burnout, generative AI, health equity, human-machine collaboration, perioperative care, preoperative distress, sociotechnical governance

## Abstract

Psychological screening is easily displaced by physiological safety checks when preoperative teams have little time and too few staff. Healthcare systems are already affected by widespread workforce shortages and clinician burnout. We suggest that preoperative anxiety consequently often remains unmanaged, with implications for postoperative recovery and hospital workload. Generic educational materials do not adequately address differences in patients’ anxiety, and “health data poverty” can further exclude vulnerable groups. We propose a conceptual human-machine collaborative (HMC) generative artificial intelligence framework to address these clinical and occupational problems together. In the proposed doctor–patient–AI feedback loop, natural language processing would dynamically assess psychosocial risk and guide patient-specific, multimodal education, including dialect-adapted voice agents, as a source of baseline emotional support. Routine interactions would be automated under clinician supervision. Our hypothesis is that this arrangement may conserve clinicians’ limited emotional bandwidth and allow healthier “cognitive reappraisal” in place of chronic fatigue. Implementation science provides a basis for considering how lightweight AI models could be scaled across regional health networks to bring standardized psychoeducation to under-resourced rural areas. This remains a theoretical pathway. Through sociotechnical governance and “data solidarity” to fairly manage algorithmic liability, the framework is still a conceptual framework, but it is operational: it proposes a path to improve perioperative workflows and health equity in the face of severe human resource constraints.

## 1 Introduction

The global health system is facing a serious shortage of personnel and widespread clinician burnout (1, 2). Continuing effort-reward imbalance (ERI) helps to explain this crisis: clinicians repeatedly undertake administrative tasks and consume emotional resources, and cumulative work requirements lead to cognitive fatigue and moral injury (3). We believe that this deficiency is particularly evident in surgical preoperative care; compact schedules make it difficult for overburdened teams to carry out psychological assessments because they can only give priority to physical safety checks. About 25 to 80% of patients have preoperative anxiety, but this estimate depends on the clinical population studied and the way in which anxiety is measured (4). We believe that when time pressure makes systematic psychological assessment impossible, a large number of such cases of anxiety are likely to be unrecognized. This is often followed by longer hospital stays and increased consumption of medical resources, which also brings further emotional stress to already exhausted clinicians (5). We propose integrating generative AI into clinical workflows as a strategic response at the public health level. Because these interventions can be scaled, they may reduce clinician burnout as well as the pressure on resources created by patient anxiety (6, 7).

## 2 The hidden burden: transforming the definition of pre-operative distress

In the case of time constraints and insufficient personnel, the traditional “one-size-fits-all” education model has become more difficult to implement (4). When the preoperative concerns of different patients are significantly different, the help of standardized materials is also very limited (8). In our view, this means that we need to rethink the meaning of “comfortable care”: psychological well-being should be considered as a core health outcome in clinical practice, and should not be considered only after the fact. Clinical communication studies have revealed this problem. Under the systemic time pressure, clinicians in a high-pressure environment give priority to the transmission of transactional medical information, pay less attention to psychological needs, often fail to provide empathetic validation, and fail to listen actively (9). Because the clinicians cannot fully respond, the patient may then conceal their anxiety. We argue that their distress often remains unaddressed. Structural power imbalances and fear of damaging the provider-patient relationship are also reasons why vulnerable patients passively withhold safety concerns, as a systematic review has shown (10). Where understaffing prevents tailored psychological support, persistent anxiety tends to delay recovery and aggravate postoperative pain; this has been well documented in pediatric surgery (5). More healthcare is consumed, admissions are longer and acute care services face additional pressure. Where stretched clinical teams leave gaps in emotional support, explainable AI, including interactive voice agents, provides a scalable means of addressing those gaps early (1). Routine anxiety is thereby prevented from obstructing clinical operations; patients’ autonomy is also safeguarded when treatment decisions are shared (11).

## 3 The doctor-patient-AI feedback loop

### 3.1 Dynamic NLP stratification and the full-cycle perioperative timeline

Figure 1 presents the AI-mediated doctor–patient–AI feedback loop that we propose for this gap in care. Generative conversational AI would provide ongoing assessment upstream of surgery, in place of relying on a conventional static screening questionnaire applied in the same way to every patient. Routine encounters supply unstructured speech and text. Natural language processing (NLP) analyzes these data so that each patient’s psychosocial risk profile can be stratified dynamically. Inviting patients to speak and encouraging them proactively are established ways of improving engagement when clinicians do so consistently. Staffing constraints, however, prevent these invitations from being provided systematically (10). In the proposed intervention, generative AI automates the structured invitations so that patients have the encouragement needed to disclose distress and clinicians have no additional workload. The assessment is arranged in two sequential preoperative phases, based on the principles of psychological prehabilitation (4, 12). Phase I would take place in ambulatory care 7 to 14 days before hospital admission. It is a baseline assessment of early anticipatory anxiety during the move from outpatient consultation to scheduled surgery (12). Once the patient is in hospital, Phase II would use targeted conversational prompts 24 to 48 h before surgery to address acute preoperative panic (8, 12). In the traditional Enhanced Recovery After Surgery (ERAS) program, continuous patient education places high demands on the organization and coordination of multidisciplinary teams. The introduction of generative artificial intelligence (AI) can digitize these communication links that consume a lot of resources. The intervention maintains the continuity of care over time, which is consistent with the evidence-based view of ERAS: surgical recovery is an active, multi-stage process that extends from preoperative preparation to postoperative rehabilitation.

**Figure 1:**
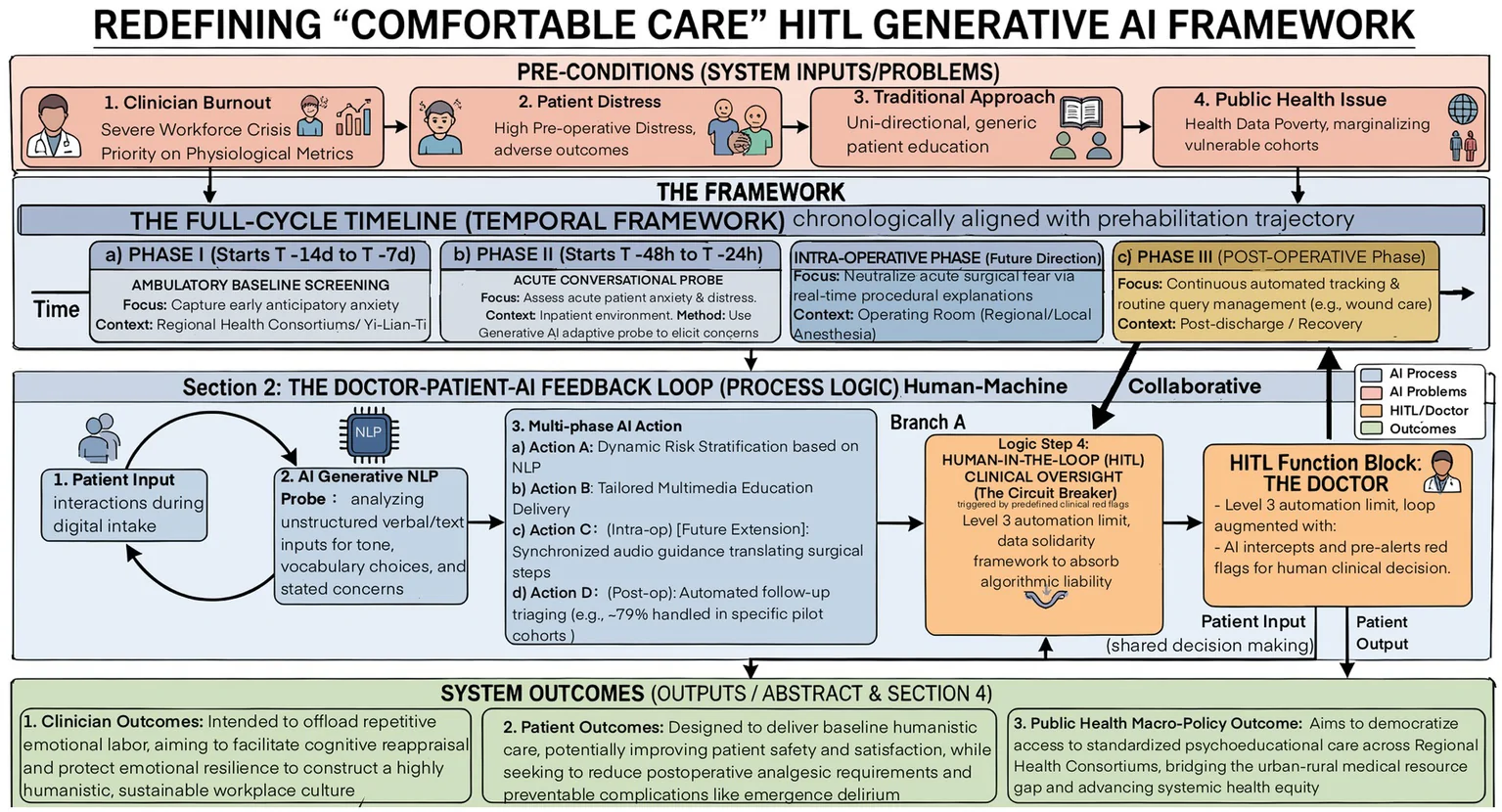
Redefining “comfortable care” HITL Generative AI framework.

In terms of patient education, we recommend perioperative pain management protocols that have been clinically approved and manually reviewed. After completing the evaluation at a specified time point, the AI model will convert these protocols into multimedia materials that are easy to use by patients, such as voice guidance adapted to the patient’s dialect and simplified cartoon infographics. Its purpose is to provide basic humanistic support without increasing the workload of clinicians (4). Systematic review evidence shows that structured procedural information and positive cognitive suggestions can directly reduce acute clinical pain (13). Automating the transmission of these specific pieces of information can provide a wider range of pain-relieving expectational cues and make up for the communication gap when clinical time is insufficient. It is particularly important to establish a risk profile early in the care path, because patient characteristics have a weak predictive ability for individual anxiety. A cross-sectional survey of 3,087 adults found that independent predictors, including female sex and previous anesthetic experience, only explained less than 13% of the variance in the anxiety score of the Amsterdam Preoperative Anxiety and Information Scale (APAIS) (8). This finding supports individualized assessment, but does not prove that generative AI is superior to static tools. The comparison between the two still needs empirical validation. Frontline clinicians generally expect the main value of AI to be relief from routine administrative and educational tasks, leaving more cognitive capacity for face-to-face care (14). Transparent disclosure of medical information has recently been identified as a strong independent predictor of patient satisfaction (9). Providing routine information by generative AI can enable clinicians to invest their saved cognitive capacity in more detailed and empathetic care. Evidence from emergency non-trauma surgery also illustrates the importance of timely identification of risks: up to 42.1% of complications are considered preventable, with the most common cause being delays in diagnosis or treatment (15). These are physical complications rather than psychological distress, but they suggest the urgency of improving perioperative workflow at a broader level. This feedback loop also extends beyond the operating room and into the third stage, postoperative monitoring. In a single-center prospective case series study, 26 patients undergoing hip arthroscopy used a rule- and intent-based SMS chatbot. Of the 128 questions, 101 (79%) were properly addressed. Only 31% of the questions were answered independently by the chatbot; the remaining issues that were properly addressed were referred to the care team (16). There was no control group in this study, and the chatbot did not use generative AI. Therefore, it is not clear whether the results can be extended to other surgeries or systems based on large language models (LLMs). We assume that conversational AI is not just a passive responder to questions. In theory, follow-up prompts based on clinical history and real-time adjustment to patients’ responses can support personalized postoperative rehabilitation and make symptom monitoring more detailed and continuous. Early detection of subtle clinical red flags, such as atypical pain flares or delayed wound healing, may provide critical intervention time for clinicians.

### 3.2 Macro-policy alignment and implementation science

“Healthy China 2030” prioritizes health literacy and preventive services, and the proposed interventions are consistent with the national policy priorities (17). How to implement them is another question. Methods from implementation science are needed to address the systemic barriers from conceptual models to clinical applications (18). Especially in the promotion of primary care from tertiary hospitals with sufficient resources, it is necessary to adapt to the limited infrastructure and large differences in health literacy. This applies not only to China’s rapidly developing medical alliances, or Yi-Lian-Ti, but also to other primary care settings (19).

For conversational agents, systemic bias is a concern from the beginning. Systematic reviews warn that agents can encode and perpetuate such bias in the absence of rigorous sociotechnical oversight, with disproportionate marginalization of groups defined by demographic or socioeconomic variables (20). Digital health datasets themselves systematically underrepresent vulnerable communities such as older adults and rural residents. This “health data poverty” accounts for much of the inequity (21). Differences in health literacy also matter: an AI tool that does not accommodate them can become a “digital determinant of health,” further marginalizing vulnerable patients (22). Responsible rollout of the distributed network must therefore address digital divides at the level of the individual patient. Developers should therefore avoid text-heavy interfaces premised on high health literacy. A locally adapted multimodal interface, such as a voice-enabled agent using regional dialects, can substantially reduce the barriers for people with limited digital skills. Inclusive design enables marginalized patients to participate meaningfully in shared clinical decision-making. Standardized psychoeducational support deployed widely on this basis can reduce the urban–rural gap in healthcare resources (19).

### 3.3 Sociotechnical governance and algorithmic safety

Clinicians remain responsible for empathetic communication, explanation of high-stakes risks and final informed consent in the human-machine collaborative workflow (14, 23). AI is an auxiliary tool. In order to maintain the role of AI as an auxiliary tool rather than mechanically replace human-centered care, it is necessary to establish sound sociotechnical governance in practical clinical applications. Bedside supervision alone is insufficient; responsibility for governance cannot rest solely with the individual clinician (1). One persistent problem is that present generative AI systems cannot adequately quantify predictive uncertainty. Other persistent risks include incorrect classification of risk and declining performance with temporal dataset shifts (24, 25). The problem of uncertainty is also accompanied by artificial hallucinations: information that sounds reasonable may be completely fabricated, but given in an assured tone, such as scientific literature citations that do not exist (26). Dataset shift is one of the main reasons for AI failure. When the training data no longer matches the dynamic clinical environment, the performance of the system will decline. This sometimes occurs after changes in patient demographic characteristics, unannounced electronic health record (EHR) system updates, or changes in clinical behavior (27). Improperly designed interfaces can cause further difficulties for already overwhelmed staff, as such interfaces can cause alert fatigue and automation bias. A systematic evaluation of healthcare conversational agents found that safety monitoring is generally insufficient; unconstrained natural language systems often fail to accurately identify serious health crises, or fail to escalate them for processing (20). These weaknesses require a built-in, enforceable “clinical circuit breaker” to prevent algorithms from causing harm and protect patients. Since the current LLM cannot reliably quantify uncertainty, we propose a rule-based safeguard independent of the confidence estimation of the generative model. When any of the following four conditions occurs, the automated circuit breaker will immediately stop the AI operation: predefined clinical red flags, such as dyspnea, calf swelling or purulence; signs of severe psychological distress; the patient directly asks for a human response; or AI deviates from approved clinical guidelines. At any time, the clinicians can also manually take over the system and continue the dialogue.

After the circuit breaker is triggered, AI no longer generates any reply. The transcript is sent immediately to the on-call clinician and a strict response time limit is set, such as 30 min. The clinician reviews the dialogue, adjusts the triage plan if necessary, and contacts the patient directly. The immutable audit log records each automatic trigger and human intervention. AI conversation can only be restored after the authorization of the attending provider (see Table 1).

**Table 1:** Matrix for pre-operative AI integration and clinical quality control.

Clinical dimension	Traditional unidirectional pathway	Proposed AI-augmented human-machine collaborative feedback loop
Care Continuity (Timeline)	Pre-op, intra-op and post-op management is fragmented, with phases often disconnected.	Full-cycle perioperative integration bridges care gaps seamlessly from admission to recovery after discharge.
Workforce Allocation	Nurses carry much of the repetitive baseline education, administrative intake and routine telephone follow-up (3).	AI is intended to take over routine education and triage post-op questions. In one orthopedic pilot, ~31% were resolved autonomously and another 48% appropriately escalated. Staff could use the cognitive capacity saved for complex, humanistic care (1, 3, 16).
Emotion Regulation (Clinician)	Chronic effort-reward imbalance may lead to “expressive suppression” and empathy fatigue (3, 32).	The design aims to conserve clinicians’ emotional resources and reduce occupational burnout by facilitating “cognitive reappraisal” (32).
Risk Stratification	Generalized questionnaires remain static, and psychological states are neglected when physiologic metrics alone are valued (5, 8).	Dynamic NLP-understanding of conversations is proposed for individualized psychosocial risk assessment in real time (8).
Intra-operative Experience	Regional/local anesthesia can leave patients environmentally isolated and acutely afraid of the unknown.	An unevaluated future extension would synchronize real-time audio explanations with the procedure, aiming to neutralize acute surgical anxiety and keep patients informed.
Post-operative Monitoring	Intermittent follow-ups require substantial staffing, with a risk that early complications will be detected late.	Continuous automated AI tracking would be subject to mandatory clinical oversight, so that missed algorithmic red flags can be intercepted and adverse events prevented (16).
Patient Autonomy & Literacy	Generic, text-heavy consent forms create high cognitive barriers in the context of “health data poverty” (21, 22).	Culturally appropriate multimodal dialogue, including dialect voice agents, is intended to improve algorithmic health literacy (21).
Governance & Liability	Rushed communication and misunderstanding create considerable medicolegal friction under individual consent models (28).	“Data solidarity” distributes algorithmic liability through collective responsibility. A clinical “circuit breaker,” with rule-based clinician override on predefined red flags, mitigates hallucination and dataset shift risks (18, 26–28).

A post-operative chatbot pilot illustrates why a human safety net is indispensable. Of the 10 issues that may require urgent treatment, the algorithm failed to adequately label three of them, including symptoms suggesting deep vein thrombosis and surgical site infection. In order to prevent injury, immediate human intervention is required (16). The rule-based safeguard itself also has inherent defects: subtle or atypical manifestations may be missed, and local dialects will also introduce additional speech-to-text translation errors. Even with the proposed safeguards, this limitation still exists. AI is most suitable for triage, rather than acting as an independent clinician.

Therefore, algorithmic risk should be shared fairly. Under the principle of “data solidarity,” hospitals, technology developers and administrative regulators work together to manage these risks while enabling clinical data to be used ethically to improve population health (28).

Implementation also depends on whether adaptive AI can be integrated into fragmented, legacy electronic health record systems, and whether front-line staff can overcome the resistance to the use of digital tools (18, 29). These infrastructure barriers are still very prominent. In order to ensure the long-term security of the algorithm, the regulatory authorities must continue to carry out post-market monitoring (24, 30). Similarly, with the continuous evolution of AI-assisted workflow, it is also indispensable to continuously carry out digital literacy training for medical professionals in order to maintain a stable clinician–patient therapeutic relationship (23, 31).

## 4 The cost–benefit risk–reward dilemma in resource-poor settings

For the care team, task-specific AI can not only bring direct clinical benefits to patients, but also play an occupational health role in balancing risks and rewards. Long-term understaffing perpetuates the effort-reward imbalance (ERI). Repeated preoperative educational and administrative tasks lead to the accumulation of fatigue and ultimately moral distress (3). For overwhelmed clinicians, expressive suppression often becomes the default emotion regulation strategy. It consumes cognitive resources, gradually weakens empathy, and directly contributes to occupational burnout.

The proposed arrangement to automate standardized routine preoperative education under the supervision of clinicians is intended to interrupt this cycle. Surgeons and nurses can shift limited cognitive resources from repetitive paperwork to cognitive reappraisal, which is a more adaptive emotion regulation strategy (32). This enables them to participate more constructively in complex patient interactions, protect emotional well-being, and support a humane and sustainable workplace culture. Different forms of communication play different roles. Routine procedural information directly reduces acute pain, while higher-order emotional care, including authentic practitioner empathy, mainly improves intermediate outcomes such as anxiety, self-efficacy, and therapeutic trust (13). Delegating standardized information transfer tasks to generative algorithms can leave more cognitive resources for clinicians to use in these human-driven and empathy-driven interactions; such interactions cannot be reproduced by synthesis. Delegating routine follow-up tasks can immediately bring workforce returns. In the orthopedic pilot, 12 of the 25 patients (48%) had been worried about complications and were reassured through chatbots. However, only 60% of patients agreed that chatbots alleviated their concerns; due to the lack of a control group, it was not possible to make definitive inferences about whether the chatbot reduced the number of outpatient calls or avoidable emergency visits (16). We envision the establishment of an efficient clinical filter to independently address some of the routine issues while transferring complex cases to staff (16). Being freed from these time-consuming organizational coordination tasks helps to reduce the daily effort-reward imbalance associated with fatigue. The value of task-specific AI at the population level is mainly due to how widely it can be deployed. Through cloud infrastructure, regional health consortia can make lightweight multimodal models available beyond tertiary hospitals with ample resources; China’s rapidly expanding medical alliances (Yi-Lian-Ti) are one such setting (19). Decentralized delivery enables rural clinicians with less experience to provide preoperative psychological support at the tertiary level. There is a practical reason to consider this for community ERAS programs. Sustained implementation is often difficult because rigorous multidisciplinary coordination is needed around the clock and local staffing cannot meet this demand. Scalable AI can take over the labor-intensive work of routine ERAS compliance, including standardized clinical pathway education and automated rehabilitation reminders, thus addressing the central logistical bottleneck (33). With standardized psychoeducation delivered at scale, the proposed AI-augmented loop is designed to have public health value outside individual encounters as well. It could constitute a population-level intervention to reduce urban–rural differences in medical resources and advance health equity (19).

## 5 Discussion

External validation is needed before clinical use, and it has to be rigorous enough to reflect the barriers encountered in real workflows (6). Reporting the area under the curve (AUC), or other aggregate statistics, is not sufficient on its own. Positive predictive value and other measures with clinical meaning belong in the validation report too (34). Pipeline transparency is a further requirement for developers, for which established reporting templates such as the MI-CLAIM checklist should be followed (35). We intend generative AI to ease surgical communication as a collaborative medium. The surgeon would not be replaced.

In order to achieve clinical adoption, it is necessary to continuously accumulate empirical evidence to prove that it can improve outcomes in different patient populations (36). Model performance is only a part of the evaluation; human factors are equally important. Improperly designed tools may cause “alert fatigue” in staff who have already experienced occupational burnout and aggravate their fatigue (14). The same pressure may also contribute to “automation complacency,” allowing staff to accept algorithmic recommendations without verifiable evidence (37). Medical curricula need to formally teach AI ethics and human–algorithm interaction to prepare clinicians to participate in collaboration (23, 31).

Awake patients receiving regional or local anesthesia might, in a future extension, be given real-time procedural explanations. This intraoperative assistance is not part of the core framework, and there is currently no direct clinical evidence. It needs to be developed and tested separately, because inaccurate explanations, interference with the attention of the surgical team, and privacy risks will bring additional safety concerns.

### 5.1 Roadmap for future empirical validation

We recommend conducting pragmatic randomized controlled trials based on objective outcomes to test whether the LLM-based approach is of practical value. After retrospective validation, we also need to evaluate the clinical effectiveness, rather than stop at the accuracy of the algorithm (25). Future trials should include the following:

1. Patient Experience: Patient satisfaction and anxiety should be recorded using existing tools, including the Amsterdam Preoperative Anxiety and Information Scale (APAIS) (38).
2. Provider Burden: Workload should be measured using a four-item subset of the NASA Task Load Index (NASA-TLX). This subset has been validated in a high-stress intensive care environment (39).
3. Workflow Efficiency: Standardized time-motion studies should be conducted according to the STAMP guidelines, making the data highly reproducible and comparable between different sites (40).
4. Clinical Safety: Missed red flags, circuit breaker activations and false-negative rates need close attention in rigorous tracking of safety metrics, together with delays in clinician response and any adverse events that occur downstream.
5. Health Equity: Utilization and clinical outcomes should be stratified by patient subgroup to ensure that care disparities are not inadvertently increased. Age, rural or urban residence, regional dialect and baseline digital literacy should be included. The latter should be assessed with the eHealth Literacy Scale (eHEALS) (41).

Reporting should include conventional clinical endpoints, such as rescue analgesic consumption and emergence delirium incidence, alongside these assessments (5). Current economic evaluations of AI also have methodological weaknesses that future work needs to address. Before infrastructure investment can be justified, comprehensive cost–benefit analyses should directly compare this framework with alternative strategies (29).

### 5.2 Limitations

No empirical testing of the conceptual framework proposed in this Perspective has yet been undertaken. Evidence cited here includes pediatric surgery (5), UK primary care (14), an orthopedic pilot of 26 patients using a non-generative chatbot (13) and emergency general surgery (15). These adjacent settings do not establish transferability to generative AI support for preoperative psychological needs. That transferability is uncertain. There is presently no evidence that generative AI reduces preoperative anxiety, burnout in clinicians or physical complications. Performance in rural China across regional dialects and different levels of health literacy has not been assessed, nor have medico-legal responsibility and cost-effectiveness. Prospective validation must demonstrate safety, clinical effectiveness and equity before the HMC framework is implemented clinically.

## Data Availability

The original contributions presented in the study are included in the article/supplementary material, further inquiries can be directed to the corresponding author.

## References

[1] Topol EJ. High-performance medicine: the convergence of human and artificial intelligence. Nat Med. (2019) 25:44–56. doi: 10.1038/s41591-018-0300-7 30617339

[2] Shanafelt TD West CP Dyrbye LN Trockel M Tutty M Wang H . Changes in burnout and satisfaction with work-life integration in physicians during the first 2 years of the COVID-19 pandemic. Mayo Clin Proc. (2022) 97:2248–58. doi: 10.1016/j.mayocp.2022.09.002 PMC9472795 36229269

[3] Tao Y Cheng Z Wang C Liu T Yan M Huang X . Perceived stress and psychological disorders in healthcare professionals: a multiple chain mediating model of effort-reward imbalance and resilience. Front Public Health. (2023) 11:1320411. doi: 10.3389/fpubh.2023.1320411, 38155891 PMC10753578

[4] Neskovic V. Preoperative anxiety and implications on postoperative recovery: what can we do to change our history. Minerva Anestesiol. (2018) 84:1307–17. doi: 10.23736/s0375-9393.18.12520-X 29624026

[5] Kain ZN Mayes LC Caldwell-Andrews AA Karas DE McClain BC. Preoperative anxiety, postoperative pain, and behavioral recovery in young children undergoing surgery. Pediatrics. (2006) 118:651–8. doi: 10.1542/peds.2005-2920, 16882820

[6] Bates DW Levine D Syrowatka A Kuznetsova M Craig KJT Rui A . The potential of artificial intelligence to improve patient safety: a scoping review. NPJ Digit Med. (2021) 4:54. doi: 10.1038/s41746-021-00423-6, 33742085 PMC7979747

[7] Thirunavukarasu AJ Ting DSJ Elangovan K Gutierrez L Tan TF Ting DSW. Large language models in medicine. Nat Med. (2023) 29:1930–40. doi: 10.1038/s41591-023-02448-8 37460753

[8] Eberhart L Aust H Schuster M Sturm T Gehling M Euteneuer F . Preoperative anxiety in adults-a cross-sectional study on specific fears and risk factors. BMC Psychiatry. (2020) 20:140. doi: 10.1186/s12888-020-02552-w, 32228525 PMC7106568

[9] Chen X Liu C Yan P Wang H Xu J Yao K. The impact of doctor-patient communication on patient satisfaction in outpatient settings: implications for medical training and practice. BMC Med Educ. (2025) 25:830. doi: 10.1186/s12909-025-07433-y, 40468313 PMC12135588

[10] Park M Giap TTT. Patient and family engagement as a potential approach for improving patient safety: a systematic review. J Adv Nurs. (2020) 76:62–80. doi: 10.1111/jan.14227, 31588602

[11] Precise4Q consortium Amann J Blasimme A Vayena E Frey D Madai VI. Explainability for artificial intelligence in healthcare: a multidisciplinary perspective. BMC Med Inform Decis Mak. (2020) 20:310. doi: 10.1186/s12911-020-01332-6, 33256715 PMC7706019

[12] Levett D Grimmett C. Psychological factors, prehabilitation and surgical outcomes: evidence and future directions. Anaesthesia. (2019) 74:36–42. doi: 10.1111/anae.14507, 30604423

[13] Mistiaen P van Osch M Van Vliet L Howick J Bishop FL Di Blasi Z . The effect of patient–practitioner communication on pain: a systematic review. Eur J Pain. (2016) 20:675–88. doi: 10.1002/ejp.797, 26492629

[14] Blease C Kaptchuk TJ Bernstein MH Mandl KD Halamka JD DesRoches CM. Artificial intelligence and the future of primary care: exploratory qualitative study of UK general practitioners’ views. J Med Internet Res. (2019) 21:e12802. doi: 10.2196/12802, 30892270 PMC6446158

[15] Linnebur M Inaba K Chouliaras K Low GM Mansfield N Benjamin ER . Preventable complications and deaths after emergency nontrauma surgery. Am Surg. (2018) 84:1422–8. doi: 10.1177/000313481808400943 30268169

[16] Dwyer T Hoit G Burns D Higgins J Chang J Whelan D . Use of an artificial intelligence conversational agent (chatbot) for hip arthroscopy patients following surgery. Arthrosc Sports Med Rehabil. (2023) 5:e495–505. doi: 10.1016/j.asmr.2023.01.020 PMC10123501 37101866

[17] Jiang Z Jiang W. Health education in the healthy China initiative 2019-2030. China CDC Weekly. (2021) 3:78–80. doi: 10.46234/ccdcw2021.018, 34595007 PMC8393088

[18] He J Baxter SL Xu J Xu J Zhou X Zhang K. The practical implementation of artificial intelligence technologies in medicine. Nat Med. (2019) 25:30–6. doi: 10.1038/s41591-018-0307-0, 30617336 PMC6995276

[19] Zeng W Gao T Zou G Li G Wang H. Design and operational features of medical alliances in China. NPJ Health Syst. (2026) 3:28. doi: 10.1038/s44401-026-00090-3, 42527518 PMC13336798

[20] Laranjo L Dunn AG Tong HL Kocaballi AB Chen J Bashir R . Conversational agents in healthcare: a systematic review. J Am Med Inform Assoc. (2018) 25:1248–58. doi: 10.1093/jamia/ocy072, 30010941 PMC6118869

[21] Paik KE Hicklen R Kaggwa F Puyat CV Nakayama LF Ong BA . Digital determinants of health: health data poverty amplifies existing health disparities—a scoping review. PLoS Digit Health. (2023) 2:e0000313. doi: 10.1371/journal.pdig.0000313, 37824445 PMC10569513

[22] Chidambaram S Jain B Jain U Mwavu R Baru R Thomas B . An introduction to digital determinants of health. PLOS Digit Health. (2024) 3:e0000346. doi: 10.1371/journal.pdig.0000346, 38175828 PMC10766177

[23] Char DS Shah NH Magnus D. Implementing machine learning in health care—addressing ethical challenges. N Engl J Med. (2018) 378:981–3. doi: 10.1056/NEJMp1714229, 29539284 PMC5962261

[24] Meskó B Topol EJ. The imperative for regulatory oversight of large language models (or generative AI) in healthcare. NPJ Digit Med. (2023) 6:120. doi: 10.1038/s41746-023-00873-0, 37414860 PMC10326069

[25] Kelly CJ Karthikesalingam A Suleyman M Corrado G King D. Key challenges for delivering clinical impact with artificial intelligence. BMC Med. (2019) 17:195. doi: 10.1186/s12916-019-1426-2, 31665002 PMC6821018

[26] Alkaissi H McFarlane SI. Artificial hallucinations in ChatGPT: implications in scientific writing. Cureus. (2023) 15:e35179. doi: 10.7759/cureus.35179, 36811129 PMC9939079

[27] Finlayson SG Subbaswamy A Singh K Bowers J Kupke A Zittrain J . The clinician and dataset shift in artificial intelligence. N Engl J Med. (2021) 385:283–6. doi: 10.1056/NEJMc2104626, 34260843 PMC8665481

[28] McMahon A Buyx A Prainsack B. Big data governance needs more collective responsibility: the role of harm mitigation in the governance of data use in medicine and beyond. Med Law Rev. (2020) 28:155–82. doi: 10.1093/medlaw/fwz016 31377815

[29] Wolff J Pauling J Keck A Baumbach J. The economic impact of artificial intelligence in health care: systematic review. J Med Internet Res. (2020) 22:e16866. doi: 10.2196/16866 PMC7059082 32130134

[30] Vayena E Blasimme A Cohen IG. Machine learning in medicine: addressing ethical challenges. PLoS Med. (2018) 15:e1002689. doi: 10.1371/journal.pmed.1002689, 30399149 PMC6219763

[31] Lee D Yoon SN. Application of artificial intelligence-based technologies in the healthcare industry: opportunities and challenges. Int J Environ Res Public Health. (2021) 18:271. doi: 10.3390/ijerph18010271, 33401373 PMC7795119

[32] Gross JJ John OP. Individual differences in two emotion regulation processes: implications for affect, relationships, and well-being. J Pers Soc Psychol. (2003) 85:348–62. doi: 10.1037/0022-3514.85.2.348, 12916575

[33] Mohamed A Francis D Fuad U Shrestha S Itoko T Pengas IP. Enhanced recovery after surgery (ERAS): transforming orthopedic trauma management. Cureus. (2025) 17:e99453. doi: 10.7759/cureus.99453, 41552184 PMC12810210

[34] Wiens J Saria S Sendak M Ghassemi M Liu VX Doshi-Velez F . Do no harm: a roadmap for responsible machine learning for health care. Nat Med. (2019) 25:1337–40. doi: 10.1038/s41591-019-0548-6 31427808

[35] Norgeot B Quer G Beaulieu-Jones BK Torkamani A Dias R Gianfrancesco M . Minimum information about clinical artificial intelligence modeling: the MI-CLAIM checklist. Nat Med. (2020) 26:1320–4. doi: 10.1038/s41591-020-1041-y, 32908275 PMC7538196

[36] Ghassemi M Oakden-Rayner L Beam AL. The false hope of current approaches to explainable artificial intelligence in health care. Lancet Digit Health. (2021) 3:e745–50. doi: 10.1016/S2589-7500(21)00208-9, 34711379

[37] Challen R Denny J Pitt M Gompels L Edwards T Tsaneva-Atanasova K. Artificial intelligence, bias and clinical safety. BMJ Qual Saf. (2019) 28:231–7. doi: 10.1136/bmjqs-2018-008370, 30636200 PMC6560460

[38] Berth H Petrowski K Balck F. The Amsterdam preoperative anxiety and information scale (APAIS)-the first trial of a German version. Psychosoc Med. (2007) 4:Doc01 PMC2736533 19742298

[39] Tubbs-Cooley HL Mara CA Carle AC Gurses AP. The NASA task load index as a measure of overall workload among neonatal, paediatric and adult intensive care nurses. Intensive Crit Care Nurs. (2018) 46:64–9. doi: 10.1016/j.iccn.2018.01.004, 29449130 PMC12863577

[40] Zheng K Guo MH Hanauer DA. Using the time and motion method to study clinical work processes and workflow: methodological inconsistencies and a call for standardized research. J Am Med Inform Asso. (2011) 18:704–10. doi: 10.1136/amiajnl-2011-000083, 21527407 PMC3168304

[41] Norman CD Skinner HA. eHEALS: the eHealth literacy scale. J Med Internet Res. (2006) 8:e507. doi: 10.2196/jmir.8.4.e27, 17213046 PMC1794004

